# Aberrant expression of kallikrein‐related peptidase 7 is correlated with human melanoma aggressiveness by stimulating cell migration and invasion

**DOI:** 10.1002/1878-0261.12103

**Published:** 2017-08-11

**Authors:** Tiphaine Delaunay, Lydia Deschamps, Meriem Haddada, Francine Walker, Antoninus Soosaipillai, Feryel Soualmia, Chahrazade El Amri, Eleftherios P. Diamandis, Maria Brattsand, Viktor Magdolen, Dalila Darmoul

**Affiliations:** ^1^ Institut National de la Santé et de la Recherche Médicale (INSERM) Hôpital Saint Louis Paris France; ^2^ Sorbonne Paris Cité UMRS‐S976 Université Paris Diderot France; ^3^ Department of Pathology Hôpital Bichat‐Claude Bernard Paris France; ^4^ Department of Pathology and Laboratory Medicine Mount Sinai Hospital Toronto Canada; ^5^ UPMC Univ Paris 06 IBPS UMR 8256 CNRS‐UPMC ERL INSERM U1164 Biological Adaptation and Ageing Sorbonne Universités Paris France; ^6^ Department of Medical Biosciences, Pathology Umeå University Sweden; ^7^ Clinical Research Unit Department of Obstetrics and Gynecology Technische Universität München Germany

**Keywords:** E‐cadherin, invasion, kallikrein‐related peptidase 7, melanoma, migration, proliferation

## Abstract

Members of the tissue kallikrein‐related peptidase (KLK) family not only regulate several important physiological functions, but aberrant expression has also been associated with various malignancies. Clinically, KLKs have been suggested as promising biomarkers for diagnosis and prognosis in many types of cancer. As of yet, expression of KLKs and their role in skin cancers are, however, poorly addressed. Malignant melanoma is an aggressive disease associated with poor prognosis. Hence, diagnostic biomarkers to monitor melanoma progression are needed. Herein, we demonstrate that although mRNA of several KLKs are aberrantly expressed in melanoma cell lines, only the KLK7 protein is highly secreted *in vitro*. In line with these findings, ectopic expression of KLK7 in human melanomas and its absence in benign nevi were demonstrated by immunohistochemistry *in vivo*. Interestingly, overexpression of KLK7 induced a significant reduction in melanoma cell proliferation and colony formation. Moreover, KLK7 overexpression triggered an increase in cell motility and invasion associated with decreased expression of E‐cadherin and an upregulation of MCAM/CD146. Our results demonstrate, for the first time, that aberrant KLK7 expression leads to a switch from proliferative to invasive phenotype, suggesting a potential role of KLK7 in melanoma progression. Thus, we hypothesize that KLK7 may represent a potential biomarker for melanoma progression.

**Abbreviations**
ELISA: enzyme‐linked immunosorbent assay
EMT: epithelial–mesenchymal transition
ERK1/2: extracellular signal‐regulated kinases 1 & 2
FBS: fetal bovine serum
GAPDH: glyceraldehyde‐3‐phosphate dehydrogenase
IHC/ISH: immunohistochemistry/*in situ* hybridization
IL‐1‐β: interleukin*‐*1
KLK: kallikrein‐related peptidase
MAP kinase: mitogen‐activated protein kinase
MCAM: melanoma cell adhesion molecule
MM: metastatic melanoma
MMP9: matrix metallopeptidase 9
NHEK: normal human epidermal keratinocytes
NHEM: normal human epidermal melanocytes
PARs: protease‐activated receptors
PCR: polymerase chain reaction
PM: primary melanoma
qPCR: quantitative polymerase chain reaction
RT*‐*PCR: reverse transcription PCR
TGF‐β: transforming growth factor beta
uPAR: urokinase‐type plasminogen activator

## 1 Introduction

Despite recent advances in the understanding of oncogenic mechanisms and therapeutic intervention, melanoma is the most aggressive skin cancer with poor prognosis in the metastatic stage. The development of melanoma begins with the malignant transformation of normal human epidermal melanocytes (NHEM) located within the basement membrane of the skin. Clinical and histopathological evidence suggests that melanoma develops sequentially, progressing from primary *in situ* melanomas, to invasive primary lesions, and finally to metastases (Haass and Herlyn, 2005). The outlined steps involve molecular changes that include acquisition of the epithelial–mesenchymal‐like transition (EMT‐like) associated with changes in cell surface adhesion molecules and activation of signaling pathways finally leading to cell dissemination (Haass and Herlyn, 2005). Despite extensive efforts concerning characterization of malignant melanoma, no specific molecular markers are currently available that are clearly related to the progression of this disease. In addition, it has been suggested that treatment failure is due to the heterogeneity of melanoma cells, which might be driven by microenvironmental factors (Postovit *et al*., 2006). Proteases are promising candidates for such molecular markers as they have been shown to be involved in invasion of cancer cells and metastasis due to their ability to degrade extracellular matrix components. Moreover, a growing body of evidence has identified diverse mechanisms by which proteases affect cancer progression and metastasis through complex processes that involve cleavage of cell adhesion molecules, growth factors, and cytokines (Sevenich and Joyce, 2014). Besides some other serine proteases, certain members of the kallikrein‐related peptidase (KLK) family serve as signaling molecules controlling cell functions also through specific membrane receptors, the protease‐activated receptors (PARs) (Darmoul *et al*., 2001, 2003, 2004; Gratio *et al*., 2010, 2011; Ramsay *et al*., 2008).

The KLK superfamily comprises 15 (KLK1‐KLK15) trypsin‐/chymotrypsin‐like serine proteases that are secreted into the extracellular space of a wide range of tissues and, furthermore, are also suggested to be involved in tumor progression (Borgono and Diamandis, 2004; Emami and Diamandis, 2007; Kryza *et al*., 2016). Several KLK members have been shown to be concomitantly upregulated in many cancers depicting them as valuable biomarkers to distinguish between normal and cancerous phenotypes, but also to predict the course of cancer disease and response to cancer therapeutics of patients (Yousef *et al*., 2003; Kontos and Scorilas, 2012).

One of the most intensively studied tissues for the evaluation of KLK functions is the skin. In normal skin, among the multiple KLKs detected, KLK7 (chymotrypsin‐like), KLK5 and KLK14 (both trypsin‐like) are considered as the major proteases (Fischer and Meyer‐Hoffert, 2013; Kalinska *et al*., 2016). They have been proposed to function as desquamatory enzymes by causing proteolysis of intercellular cohesive structures in the stratum corneum (Caubet *et al*., 2004). Elevated expression of KLKs – in particular KLK5 and KLK7 – has in fact been found in skin diseases involving skin barrier disorders (Fischer and Meyer‐Hoffert, 2013; Kalinska *et al*., 2016).

During tumor progression, higher levels of KLK7 in tumor tissue have been mainly associated with poor prognosis in a variety of cancer types, even when the expression level was found to be downregulated in relation to the corresponding nonmalignant, normal tissue (Devetzi *et al*., 2013; Kryza *et al*., 2016; Stefanini *et al*., 2015). Elevated KLK7 expression is, for example, correlated with short survival in colon, ovarian, and pancreatic cancers (Devetzi *et al*., 2013; Dorn *et al*., 2014; Iakovlev *et al*., 2012). So far, analysis of KLK expression levels and functions in skin cancer has not been addressed in detail. Still, using microarray technology approaches, gene network analysis, and immunohistochemistry, recent studies point to the importance of KLKs in melanoma progression. Indeed, it has been shown that KLK6, 7, 8, and 13 are co‐ordinately expressed in melanoma progression. In addition, KLK7 was found to be associated with good prognosis and survival outcome of patients with primary melanoma (Martins *et al*., 2011; Rezze *et al*., 2011). Concerning KLK6, a controversial study reported that this protein is not expressed by melanoma cells but rather by keratinocytes and stromal cells of the microenvironment (Krenzer *et al*., 2011).

In the present study, we demonstrate, for the first time, aberrant expression and secretion of multiple KLKs in melanoma cell lines. KLK7 displayed ectopic expression in melanoma cells *in vitro* and in resected tumors from patients with primary and metastatic melanomas but was absent in nevi. Furthermore, we clearly show that KLK7 overexpression in melanoma cells induces a decrease in cell proliferation and colony formation. Concurrently, a loss of E‐cadherin expression and upregulation of melanoma cell adhesion molecule (MCAM)/CD146 are observed, which are associated with an increase in cell motility and cell invasion. Thus, these data suggest that KLK7 is not only a potential biomarker for melanoma progression, but also plays a role in tumor invasion.

## 2 Materials and methods

### 2.1 Reagents

Neomycin (or G418), DMEM, RPMI 1640, and HAM's F12 medium were purchased from Life Technologies (Cergy‐Pontoise, France), and the Nucleospin RNA kit from Macherey–Nagel (Düren, Germany). Antibodies were purchased from the following vendors: human KLK7 polyclonal antibody (#GTX103548) from GeneTex Inc. (Irvine, CA, USA); E‐cadherin (32A8) (#5296) and mouse phospho‐specific antibodies to ERK1/2 (Thr202/Tyr204) (#9106) from Cell Signaling Technologies (Beverly, MA, USA); polyclonal anti‐ERK1/2 (#SC‐94) antibodies from Santa Cruz Biotechnology (Santa Cruz, CA, USA); MCAM/CD146 from R&D systems (Lille, France); peroxidase‐conjugated anti‐mouse (#115‐035‐068) and anti‐rabbit (#111‐035‐144) antibodies from Jackson ImmunoResearch (West Grove, PA, USA); and Alexa Fluor^®^ 488 anti‐mouse IgG from Invitrogen (Carlsbad, CA, USA). Purified rabbit IgG was obtained from Sigma Aldrich (Lyon, France).

### 2.2 Cell culture

Human melanoma cell lines (Colo 792, MeWo, 501Mel, A‐375, Colo 794, Colo 829, Dauv*‐*1, M74, MM170, SK‐Mel5, SK‐Mel28, WM115, WM 266‐4, C8161, HM11, SBcl2, WM1361, WM852, XP44RO, MM127, MT10, M230, and WM1791C) were kindly provided by Nicolas Dumaz (INSRM U976, France) (Dumaz *et al*., 2006). All melanoma cell lines have been genotyped to verify their authenticity. WM115 is derived from a primary melanoma, and WM266.4 – a metastatic melanoma cell line – isolated from the same patient. Cells were cultured in high‐glucose DMEM or RPMI 1640 medium supplemented with 10% fetal bovine serum (FBS), 100 U·mL^−1^ penicillin, 100 μg·mL^−1^ streptomycin (Invitrogen) at 37 °C with 5% CO_2_. Normal neonatal human epidermal melanocytes (NHEM) were from PromoCell (Heidelberg, Germany) and Cascade Biologics (Nottinghamshire, UK). They were grown in KBM‐Gold medium supplemented with stem cell factor (Lonza, Aubergenville, France) or in medium 154 supplemented with human melanocyte growth supplement (Cascade Biologics). HaCaT, immortalized keratinocytes, were cultured in DMEM supplemented with 10% FBS (ATCC, Rockville, MD, USA).

### 2.3 Reverse transcription polymerase chain reaction (RT‐PCR)

Four micrograms of total RNA was reverse‐transcribed using oligo (dT) primers and ThermoScript kit (ThermoFisher Scientific, Villebon sur Yvette, France) according to the manufacturer's instructions. Ten percent of the reaction was used as template in the PCR to amplify human KLK4, KLK5, KLK6, KLK7, KLK8, KLK10, or KLK14. Primer sequences and conditions are described in Table S1. GAPDH cDNA amplification was used as an internal control. After 35 cycles of amplification, PCR products were identified by electrophoresis in 2% agarose gels followed by SYBR^®^Safe staining (Invitrogen).

### 2.4 Quantitative reverse transcription PCR

One microgram of total RNA was reverse‐transcribed, and PCR were then performed in duplicate using the Power SYBR^®^ Green PCR Master Mix kit (ThermoFisher Scientific) following the manufacturer's instructions. The final 10 μL reaction volume included 5 μL of 2 × Master Mix, 50 nm of each primer, and 2 μL cDNA. The following primers were used: 5′CCCAGTGCTCTGAATGTCAA3′ (forward) and 5′AGTGGGAATCTCGTTCATCC3′ (reverse) for KLK7; 5′TGGGTGTGAACCATGAGAAGTATG3′ (forward) and 5′GGTGCAGGAGGCATTGCT3′ (reverse) for GADPH, a housekeeping gene used as an internal standard. The QPCR conditions were as follows: 10 min at 95 °C, then 40 cycles of amplification at 95 °C for 15 s and 60 s at 60 °C. Relative quantification of the target gene expression was performed using the comparative cycle threshold (*C*_t_) method (Sequence Detection Systems 2.0; Applied Biosystems). *C*_t_ was normalized to GAPDH (Δ*C*_t_ = *C*_t_ sample − *C*_t_ GAPDH).

### 2.5 KLK ELISA assays

Melanoma cells were seeded at 40 000 cells/well in 12‐well plates. At confluence, the conditioned medium was collected for antigen determination of various KLKs using a noncompetitive immunoassay as previously described (Shaw and Diamandis, 2007) (Gratio *et al*., 2011), whereas the cells were detached and counted.

### 2.6 Tissue immunohistochemistry

Immunohistochemistry was performed on formalin‐fixed, paraffin‐embedded tissue samples (*n* = 38) encompassing six cases with nevi, 18 cases with primary melanoma, and 14 cases with metastasis (Pathology Department of Bichat‐Claude Bernard Hospital, Paris). Tissues were analyzed in accordance with the requirement of the Human Research Committee of the Bichat‐Claude Bernard Hospital and according to the Declaration of Helsinki as adopted by the French Public Health Code ([Link]). Tumors were staged according to the seventh International Union Against Cancer (UICC). KLK7 immunostaining was performed using a Leica Bond Max Automated IHC/ISH Stainer and the Bond Polymer Refine Detection Kit (Leica Microsystems Inc., Nanterre, France) according to the manufacturer's instructions. Briefly, the machine performs all steps of deparaffinization, heat‐induced antigen retrieval in high‐pH (pH, 9) bond retrieval solution at 100 °C. After endogenous peroxidase quenching, anti‐human KLK7 (N1C3) polyclonal antibody or rabbit IgG control was applied both at a 1 : 100 dilution. After incubation, post‐primary antibody and thereafter, poly‐HRP‐IgG reagent were added. Detection was performed using DAB (3,3′‐diaminobenzidine tetrahydrochloride) reagent. Nuclei were counterstained with Mayer's hemalum solution. Specificity of KLK7 immunostaining has been demonstrated by rabbit IgG (data not shown) and by omitting the primary antibody (Walker *et al*., 2014). Staining was assessed by three independent observers employing a semiquantitative methodology: First, the percentage of KLK7‐immunostained cancer cells was evaluated and second, the staining intensity was scored on a scale between no (0), weak (+1), moderate (+2), and strong (+3) staining.

### 2.7 Western blot analysis

Recombinant human proKLK7 was produced and purified from yeast cells as described earlier (Stefansson *et al*., 2008). The enzyme was then activated using thermolysin, and the activity was routinely tested against a fluorogenic substrate (data not shown). Quiescent cells were treated with recombinant active recombinant KLK7 at different concentrations and for various time periods as indicated in the Results section. Cells were lysed with RIPA buffer as described (Darmoul *et al*., 2004). Equal amounts of extracts (25 μg) were separated by SDS/PAGE and transferred onto a nitrocellulose membrane. Membranes were incubated in blocking buffer (20 mm Tris, 50 mm NaCl) containing 5% (w/v) low‐fat milk and 0.1% (v/v) Tween 20 and then probed with a monoclonal phospho‐specific antibody directed to ERK1/2 (1 : 2000) overnight at 4 °C. Membranes were stripped in stripping buffer (Invitrogen) and reprobed with a polyclonal anti‐ERK1/2 antibody (1 : 1000) that recognizes total ERK1/2 regardless of its phosphorylation state as loading controls. Proteins were revealed applying the Signal^®^ Chemiluminescent Substrate (Thermo Scientific) on an Image Quant imaging system.

### 2.8 Transfection of the KLK7 expression plasmid and selection of stable transfectants

**Figure 1:**
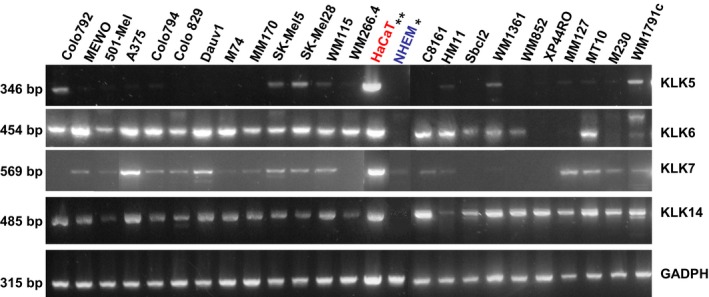
Analysis of the mRNA expression pattern of KLK5, 6, 7, and 14 in different melanoma cell lines by RT‐PCR. Analysis was performed using RNA isolated from the indicated human melanoma cell lines. mRNA expression of KLK5, 6, 7, and 14 was characterized by semiquantitative PCR as described in the Materials and methods section.*Normal human embryonic melanocytes (NHEM), used as negative control; **human keratinocyte‐like HaCaT cells, used as positive control; and GADPH as internal control.

The cDNA encompassing the coding region of KLK7 was inserted into the mammalian expression vector pRc/RSV (Invitrogen) yielding pRc/RSV‐KLK7 as described (Prezas *et al*., 2006). pRc/RSV (vector control) and pRc/RSV‐KLK7, respectively, were transfected into the KLK7‐deficient melanoma cell line M74 (see Fig. 1), using the Lipofectamine 3000 reagent (Invitrogen) according to the manufacturer's instructions. Forty‐eight hours after transfection, the culture medium was replaced by selective medium containing G418 (1 mg·mL^−1^). The batch of neoresistant pRc/RSV‐KLK7‐transfected M74 cells were denoted as M74‐H, a selected clone, which expressed high amounts of KLK7, as M74‐D6. M74 cells, stably transfected with the vector only, were designated as M74‐mock cells.

The catalytic triad serine to alanine mutant‐KLK7‐S195A plasmid was generated by site‐directed mutagenesis (Fig. S1). In the set of experiments, designed for analysis of the active site mutant, M74 cells transfected with KLK7 mutant (KLK7‐S/A) cDNA were denoted M74‐KLK7‐S/A.

### 2.9 Cell proliferation assay

Cell proliferation was determined either by direct cell counting or using the WST‐1 Cell Proliferation Assay Kit (Roche Diagnostics GmbH, Mannheim, Germany).

For direct counting, cells were seeded at a density of 2 × 10^4^ cells (48‐well plates) and incubated at 37 °C for 24, 48, 72, or 96 h. Cells were detached from triplicate wells and counted in a hemacytometer. Cell death was evaluated by staining of the cells with trypan blue. At least three independent experiments were performed for each experimental condition.

For the WST‐1 assay, cells were seeded at a density of 5 × 10^3^ cells (96‐well plates) and incubated at 37 °C for 24, 48, 72, or 96 h. WST‐1 was added to each well and the plate was incubated in 5% CO_2_ at 37 °C for 2 h. Plates were finally analyzed by measuring the absorbance at 450 nm *versus* the reference wavelength of 630 nm using a scanning multiwell spectrophotometer. Three independent experiments were performed for each experimental condition.

### 2.10 Clonogenic assay

To test the ability of single cells to grow into a colony, KLK7‐expressing cells (M74‐D6 and M74‐H) or vector control cells (M74‐mock) were plated at a low density (1000 cells/well) in six‐well plates and allowed to generate single colonies for 14 days. The colonies were washed twice in PBS, then stained with 0.5% (v/v) crystal violet/20% methanol, imaged, and quantified using an Image Quant™ LAS 4000 digital imaging system and the image j software (GE Healthcare, Piscataway, NJ, USA). At least three independent experiments were performed in duplicate.

### 2.11 Immunofluorescence staining

E‐cadherin and MCAM/CD146 immunofluorescence detection was performed with cells grown on glass coverslips (IBD). Cells were washed three times in PBS, fixed in 2% paraformaldehyde, washed three times in PBS, and then incubated with PBS containing 2% BSA for 15 min prior to application of the primary anti‐E‐cadherin or anti‐MCAM/CD146 antibodies (1 : 200) for 2 h at room temperature. Subsequently, cells were incubated for 45 min with the secondary antibody goat anti‐mouse IgG coupled to Alexa‐488 Fluor. Negative controls were obtained by omitting primary antibodies. Finally, the cells were mounted in Vectashield medium containing DAPI Dye (Vector, Peterborough, UK) and examined using a fluorescence microscope (Zeiss, Jena, Germany).

### 2.12 Cell migration and Matrigel™ invasion assay

For the *in vitro* cell migration assay, 8‐μm pore‐size Transwell^®^ inserts (Ibidi, Martinsried, Germany) were used according to the manufacturer's instructions. The chambers were placed into 24‐well dishes containing 750 μL of RPMI medium supplemented with 10% FBS as a chemoattractant. Cells (2 × 10^4^) were added to the upper well of each chamber in 200 μL of serum‐free RPMI medium.

For the cell invasion assay, Transwell^®^ inserts were coated with 10 μg of Matrigel™ (Biocoat; BD Biosciences, San Jose, CA, USA) in 100 μL of RPMI at 37 °C. The coated chambers were air‐dried for 6 h. The chambers were then placed into 24‐well dishes containing 750 μL of RPMI medium supplemented with 10% FBS. Cells (5 × 10^4^) were seeded into the upper chamber of the device in 200 μL of serum‐free RPMI medium. After a 24‐h incubation period, migrated or invaded cells on the lower side of the insert were stained with a 0.5% (v/v) crystal violet/20% methanol solution and imaged by bright field microscopy in six random fields at magnification × 4. The number of stained cells after migration or invasion was counted with the image j software.

### 2.13 Statistical analysis

Data show the mean values of at least three independent experiments. The GraphPad Prism statistical tool was used to perform the one‐way ANOVA. A *P*‐value < 0.05 was considered statistically significant (^NS^> 0.05, *P <* *0.05, **P <* *0.01, ***P <* *0.001).

## 3 Results

### 3.1 Kallikrein‐related peptidases are ectopically expressed in human melanoma cell lines

As recent studies point to the importance of KLKs in melanoma progression (Martins *et al*., 2011; Rezze *et al*., 2011), we aimed at investigating the expression pattern of KLKs in melanoma cells. mRNA levels of several KLKs were analyzed by RT‐PCR in 23 human melanoma cell lines. We first focused on KLK5, KLK6, KLK7, and KLK14 that have been shown to play major physiological roles in the skin (Fischer and Meyer‐Hoffert, 2013; Kalinska *et al*., 2016). As shown in Fig. 1, KLK5, KLK6, KLK7, and KLK14 mRNA were expressed in 34%, 86%, 69%, and 100% of the analyzed cell lines, respectively. Also other KLKs – KLK4, 8, and 10 – were found to be present in 30%, 13%, and 39% of melanoma cell lines, respectively (for a summary of the expression pattern of all analyzed KLKs, see Table 1). In contrast, all *KLKs* tested were absent or only very weakly expressed in normal human melanocytes (NHEM) (Fig. 1). As expected, keratinocyte‐like HaCaT cells, taken as a control, express high levels of the *KLK* genes tested in our study (Lundwall and Brattsand, 2008). Thus, our data suggest that melanoma cell lines ectopically express certain *KLK* genes that are absent or very weakly expressed in normal melanocytes. We did not find major differences in the *KLK* expression patterns of the primary melanoma cell lines (i.e.*,* WM115, Dauv1, WM1361, HM11, and SBcl2) as compared to metastatic cell lines (Tables 1 and S2). In addition, no correlation was found between expression of *KLKs* and BRAF, NRAS or C‐Kit mutations (Table S2), which are the most frequently mutated oncogenes in melanomas (Flaherty *et al*., 2012).

**Table 1:**
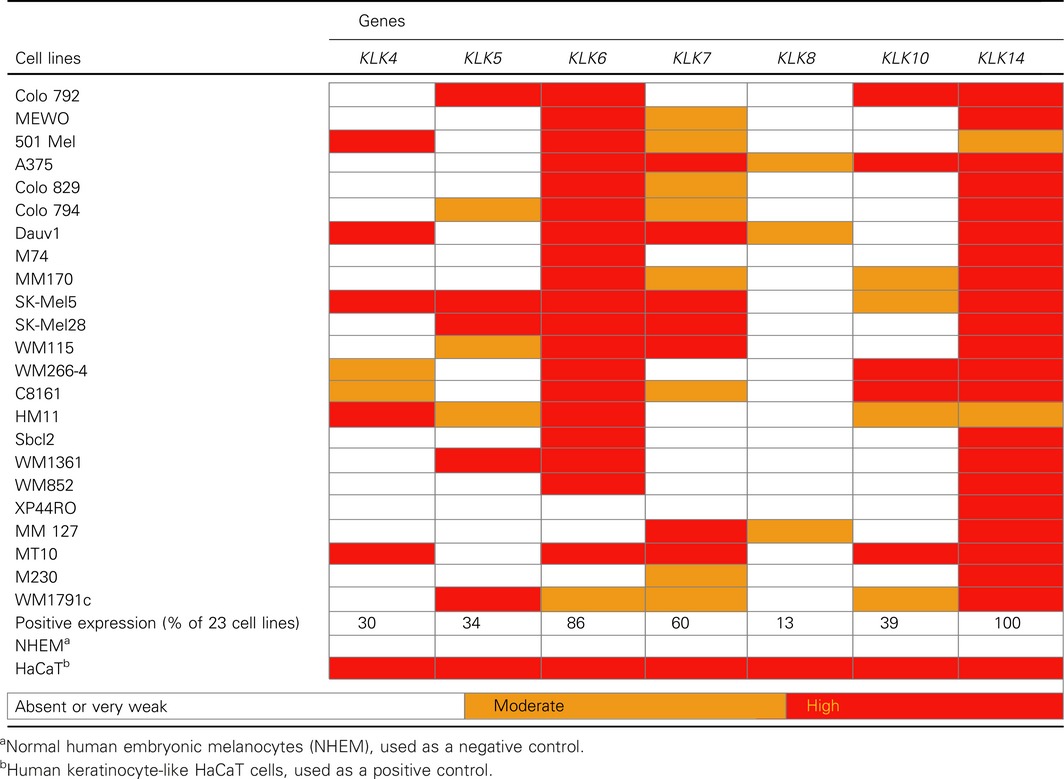
KLK mRNA expression levels in human melanoma cell lines analyzed by semiquantitative RT‐PCR

### 3.2 Detection of secreted KLKs in supernatants of human melanoma cells

**Figure 2:**
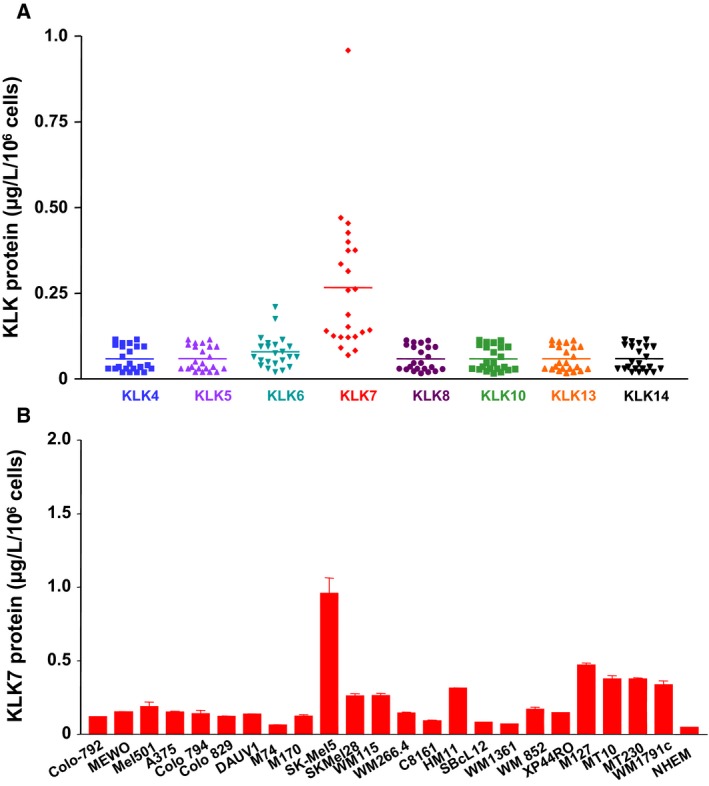
Quantification of the antigen levels of various KLKs in the cell culture supernatants of human melanoma cell lines by ELISA. (A) Secretion of KLK 4, 5, 6, 7, 8, 10, 13, and 14 in conditioned medium from 23 melanoma cell lines was assessed by ELISA and is presented in a scatter plot. Protein values represent the mean concentration of KLKs (μg·L^−1^) secreted by 10^6^ cells, which were cultured for 24 h. (B) Conditioned medium was collected from 23 melanoma cell lines and KLK7 concentration assessed by ELISA. Protein values represent the mean concentration of KLK7 (μg·L^−1^) secreted by 10^6^ cells, which were cultured for 24 h. Note that normal melanocytes (NHEM cells) express very low, if any, KLK7.

Protein expression of various KLKs was investigated by measuring secretion of these proteins into the conditioned media from 23 human melanoma cell lines and from normal melanocytes. The concentrations of KLK4‐8, KLK10, KLK13, and KLK14 in the cell line supernatants were quantified using immunoassays as previously described (Gratio *et al*., 2011; Shaw and Diamandis, 2007). As shown in Fig. 2, many KLKs were detected in the supernatants. However, mRNA levels did not in all cases reflect the protein levels (compared to Table 1), a common phenomenon due to the complexity of the transcriptional regulation (Kryza *et al*., 2016). Interestingly, KLK7 was the major secreted KLK by human melanoma cell lines (Fig. 2A). Highest KLK7 levels (958 ± 106 ng·L^−1^/10^6^ cells; ~ 38 pmol·L^−1^) were observed in the conditioned media from SK‐Mel5 cells followed by M127, MT10, M230, WM1791c, HM11, SK‐Mel28, and WM115 cells (Fig. 2B). In accordance with the PCR results, KLK7 immunodetection in normal melanocytes (NHEM) as well as in the other analyzed cell lines was very low or below the detection limit of the current protocol. Subsequent quantitative PCR analysis confirmed that SK‐Mel5, A375, MeWo, Dauv1, SK‐Mel 28, WM115, and MT10 cells display the highest *KLK7* mRNA levels, whereas NHEM cells, obtained from two different sources (Fig. S2), do not express KLK mRNA. Taken together, these data suggest that melanoma cell lines express and secrete distinct amounts of KLKs that could potentially act in an autocrine manner and play a role in melanoma progression.

### 3.3 KLK7 expression in primary and metastatic melanomas *in vivo*

**Figure 3:**
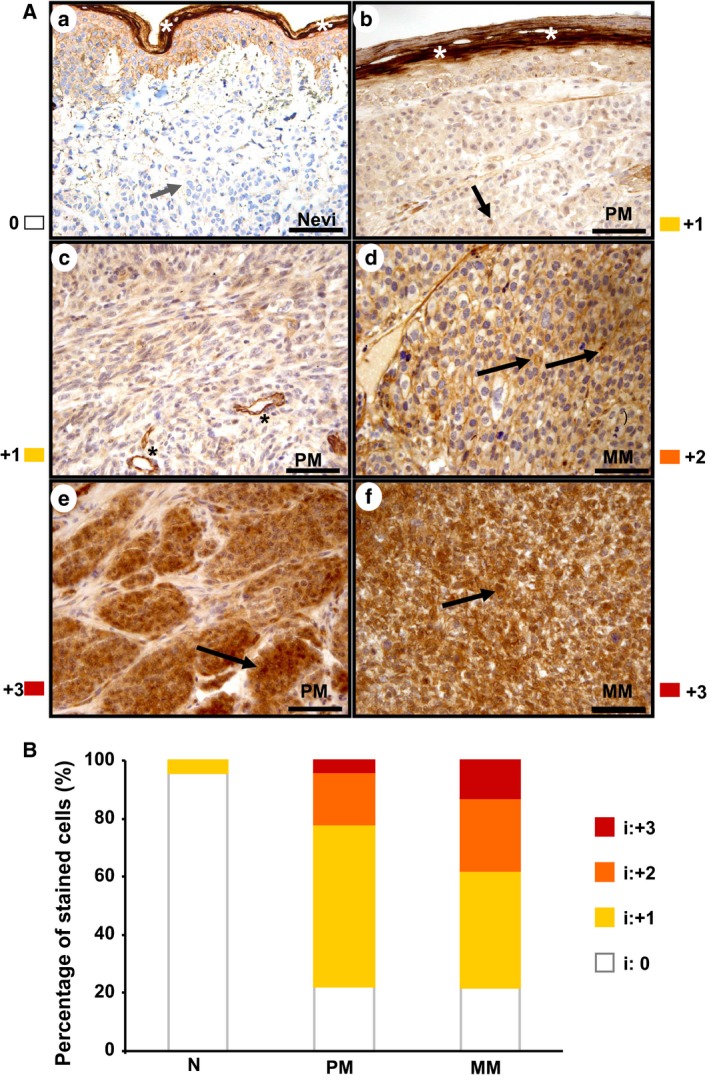
Detection of KLK7 on formalin‐fixed, paraffin‐embedded sections of primary and metastatic melanomas by immunohistochemistry. (A) Representative images of the KLK7 expression pattern in human melanocytic lesions obtained by immunohistochemical staining. (a) Nevi with negative staining (intensity: 0); (b, c) primary melanoma with weak KLK7 staining (intensity: +1); (d, f) metastatic melanoma with moderate to strong KLK7 staining (intensity: +2 and +3); (e) primary melanoma with strong KLK7 staining (intensity: +3). Note that there is no staining in the nevi, but a significantly increased staining in primary (PM) and metastatic melanoma (MM). Immunohistochemical staining generally exhibits a heterogeneous cytoplasmic KLK7 expression regardless of the stage (compare b, c, and e *versus* d and f). As expected, normal‐appearing epidermal cell layers adjacent to nevi (a) or to primary melanoma (b) show the highest KLK7 levels in the stratum corneum layers (white stars) (Yousef *et al*., 2000). Black arrows in (b), (d), (e), and (f) point to positive KLK7 immunoreactivity in the cytoplasmic compartment of the cells. The gray arrow in (a) points to a normal melanocyte with negative staining. Black stars in (c) indicate KLK7 staining in the vessels. Scale bar = 200 μm. (B) Staining intensity of KLK 7 was categorized into intensity ranges from 0 to 3+. The average percentage of cells in each group was determined and is shown in a stacked graph for each tissue type (*n* = 38). N, nevi; PM, primary melanoma; MM, metastatic melanoma.

To validate ectopic expression of KLK7 in melanoma *in vivo*, we next examined the pathological relevance of our observations by performing immunohistochemistry of KLK7 in primary and metastatic melanomas *versus* nevi. As shown in Fig. 3, KLK7 was not or barely detected in nevi, but clearly elevated in primary and metastatic melanomas. Staining was localized to the cytoplasmic compartment of the cells. Normal‐appearing epidermal cell layers adjacent to nevi (Fig. 3A‐a) or to primary melanoma (Fig. 3A‐b) showed the strongest KLK7 staining in keratinocytes, in concordance with its described high‐level expression in the stratum corneum layers (Egelrud *et al*., 2005). In both primary and metastatic melanomas, the intensity of KLK7 staining varied in the different patient samples analyzed and was evaluated as weak (Fig. 3A‐b,c), moderate (Fig. 3A‐d), and strong (Fig. 3A‐e,f), respectively. Interestingly, analysis of the intensity of KLK7 immunoreactivity revealed that metastatic melanoma tissues showed a higher average of cells with strong intensity compared to primary melanoma (13.5% *versus* 3.5%). The number of cells with moderate intensity was also higher in metastatic melanoma samples compared to primary melanomas (25% *versus* 18%), while the average number of cells with weak intensity staining was higher in primary melanomas compared to metastatic melanoma samples (42% *versus* 56%) (Fig. 3B). No specific staining was seen when the primary antibody was replaced with the rabbit IgG (not shown). These observations show that human melanoma cells aberrantly express high levels of KLK7 in contrast to benign melanocytes or benign nevi. This suggests that KLK7 expression may represent a potential marker for melanoma progression.

### 3.4 KLK7 enhances ERK1/2 activation in human melanoma cell lines

**Figure 4:**
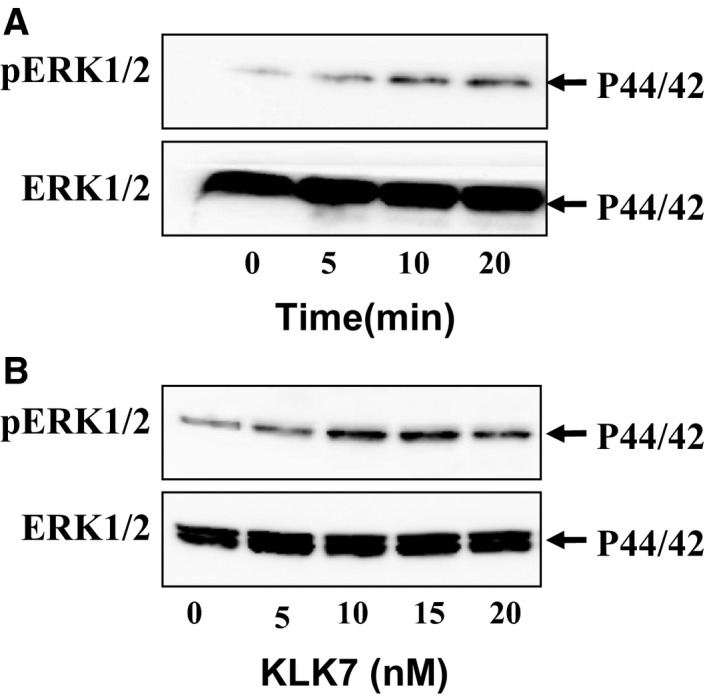
KLK7 triggers ERK1/2 phosphorylation in melanoma cells. (A) Immunoblot detection of phosphorylated ERK1/2 (upper panel) in quiescent MeWo cell lysates treated with or without KLK7 (10 nm) for the indicated time periods. To confirm equal protein loading, the membranes were stripped and incubated with an antibody detecting both nonphosphorylated and phosphorylated ERK1/2 (lower panel). Results are representative of two separate experiments. (B) Dose‐dependent activation of ERK1/2 phosphorylation by KLK7. Quiescent MeWo cells were stimulated with the indicated concentrations of KLK7 for 10 min. The upper panel shows the result obtained with an antibody specifically directed to phosphorylated ERK1/2, and the lower panel the result using an antibody detecting both nonphosphorylated and phosphorylated ERK1/2. Results are representative of two separate experiments.

Because KLK7 has been proposed to act as a proliferative factor in many cancer types (Kryza *et al*., 2016; Walker *et al*., 2014), we next investigated the effect of KLK7 on mitogen‐activated protein kinase (MAP kinase) (ERK1/2) phosphorylation, an upstream kinase in the proliferation pathways. Addition of KLK7 (10 nm) to quiescent MeWo cells, at various time points, induced a rapid and significant phosphorylation of ERK1/2, reaching a maximum within 5–10 min (Fig. 4A). In addition, KLK7 induced ERK1/2 phosphorylation in the range between 5 and 20 nm, whereby maximum ERK1/2 phosphorylation was obtained already with 10 nm KLK7 (Fig. 4B). These experiments suggest that KLK7 activates the MAP kinase pathway in melanoma cells, possibly playing an important role in melanoma proliferation.

As members of KLKs have been suggested to modulate cytokine and TGF‐β in some cancer types (Kryza *et al*., 2016), we also analyzed phosphorylation of Smad 2 (TGF‐β pathway), Stat3 (cytokine and growth factor pathways) in two different *in vitro* cell models; (a) in KLK7‐overexpressing melanoma M74 cells (upon stable transfection with a KLK7 expression vector, see section below), grown under serum‐free conditions, and (b) in Mewo cells, stimulated with recombinant KLK7 under serum‐free conditions. In neither of the models, an evidence for a KLK7‐mediated modulation of cytokine, growth factor, or TGF‐β pathways was found (Fig. S3).

### 3.5 KLK7 overexpression induces reduction in melanoma cell growth and colony formation

**Figure 5:**
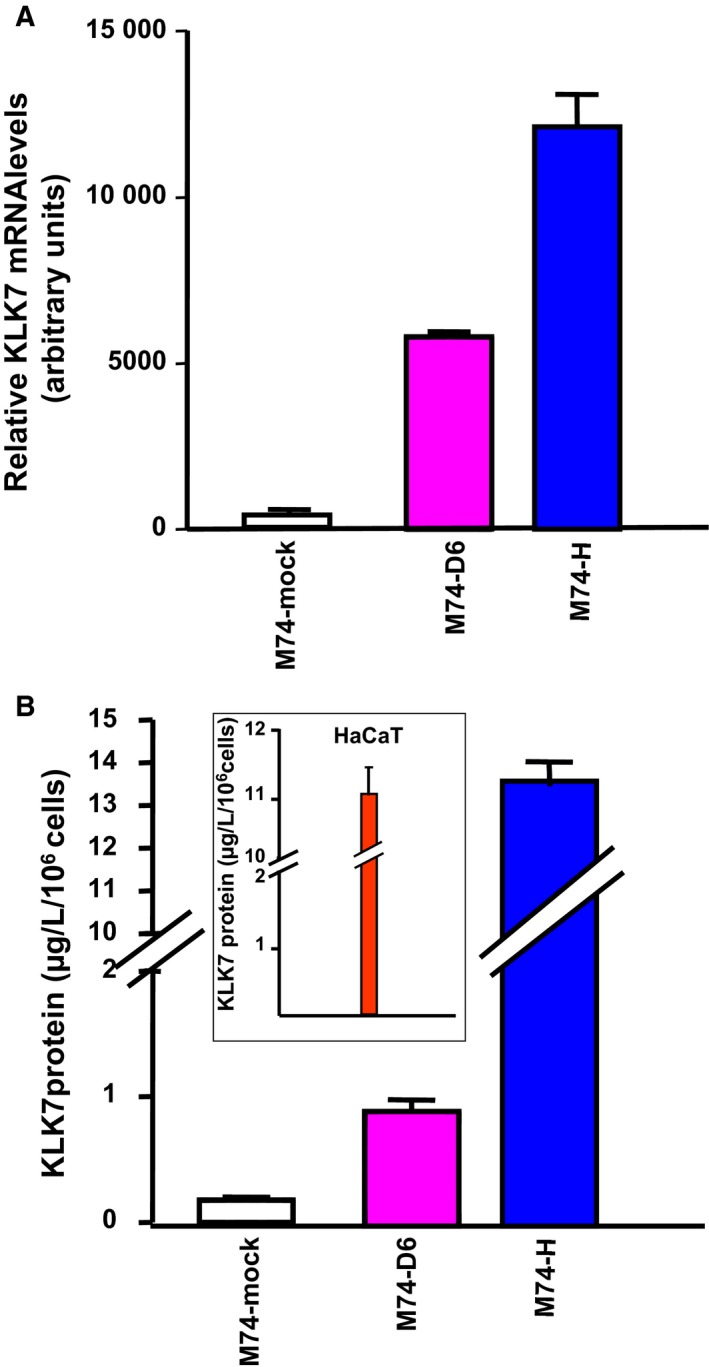
Quantification of KLK7 mRNA and measurement of KLK7 protein secretion in melanoma M74‐derived cell lines. Melanoma M74 cells were stably transfected with the KLK7 expression plasmid pRcRSV‐KLK7 or with the pRcRSV vector alone. M74‐D6 represents a selected clone overexpressing KLK7, and M74‐H is a batch‐transfected M74‐derived KLK7‐overexpressing cell line. M74‐mock, M74 cells, stably transfected with the empty vector only. (A) Total RNA from either M74‐D6, M74‐H, or M74‐mock cells was reverse‐transcribed and subsequently analyzed by SYBR Green fluorescence‐based QPCR to quantify the KLK7 mRNA expression levels. *GAPDH* was used as the endogenous reference gene for normalization of KLK7 expression. (B) Conditioned medium was collected from either M74‐D6, M74‐H, or M74‐mock cells after 24 h, and KLK7 antigen levels were assessed by ELISA. The inset depicts the KLK7 antigen levels measured in cell culture supernatants of HaCaT cells. Data represent the mean protein values ± SEM of three independent experiments.

To examine the possible function of KLK7 in melanoma cells, we stably overexpressed KLK7 in the M74 melanoma cell line, using the KLK7 expression vector pRcRSV‐KLK7. M74 cells were chosen because in these cells expression of KLK7 is very low (see Fig. 1 and Table 1). We tested both a selected clone (M74‐D6) and batch‐transfected cells (M74‐H). As shown in Fig. 5A, QPCR analysis revealed that the selected M74‐D6 clone as well as the batch‐transfected cells M74‐H expresses significant amounts of KLK7 mRNA. As expected, the vector‐transfected control cells (M74‐mock) did not express KLK7 mRNA. Moreover, ELISA measurements showed that in M74‐D6 and M74‐H cells, the KLK7 protein was also secreted into the cell culture medium (Fig. 5B). Notably, the secreted KLK7 levels in M74‐H cells were comparable to those secreted by HaCaT keratinocyte‐like cells (Fig. 5B, inset).

**Figure 6:**
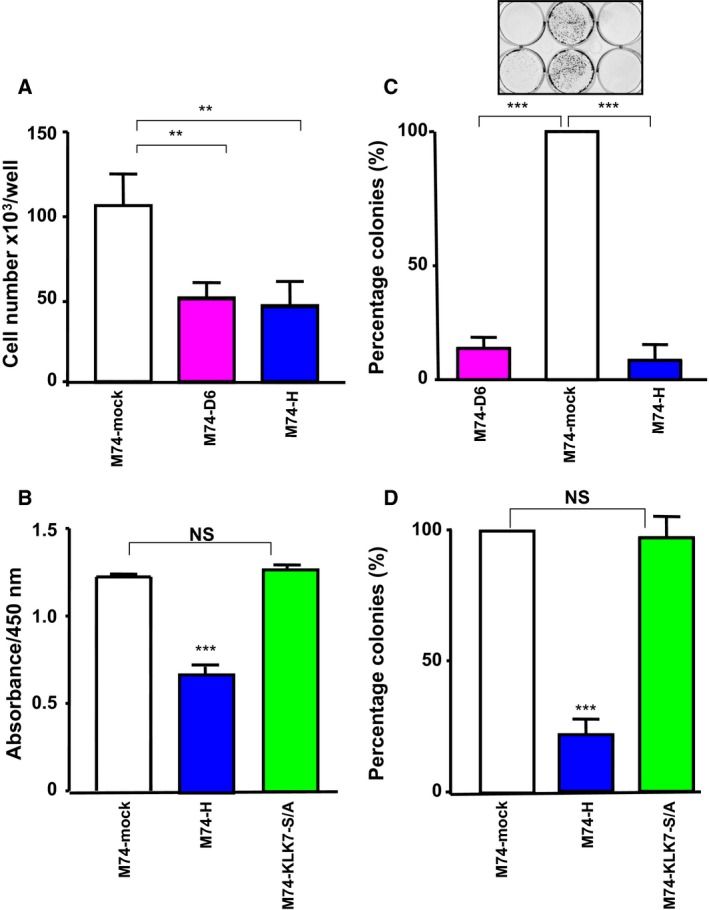
Overexpression of wild‐type KLK7, but not the active site mutant KLK7‐S/A, inhibits cell growth and colony formation in melanoma cells. (A) M74‐D6, M74‐H, and M74‐mock cells were seeded as described in Materials and methods and counted after 96 h. Data are given as mean ± SEM of three independent experiments. **P <* *0.01, M74‐D6 and M74‐H cells *versus* M74‐mock cells. (B) M74‐cells overexpressing wild‐type KLK7 (M74‐H), or its active site mutant (M74‐KLK7‐S/A), or M74‐vector cells (M74‐mock) were seeded as described in Materials and methods, and proliferation was analyzed after 72 h using the WST‐1 Cell Proliferation Assay Kit. Data are given as mean ± SEM of three independent experiments. M74‐H *versus* M74‐mock, ***P <* *0.001; M74‐KLK7‐S/A *versus* M74‐mock, NS (*P* > 0.05). (C) M74‐D6, M74‐H, and M74‐mock cells were plated as described in Materials and methods and incubated for 2 weeks. KLK7 induced a strong decrease in melanoma colony formation. Representative images (upper panel) were captured and quantified (lower panel) with an Image Quant™ LAS 4000 digital imaging system. Columns represent the mean percentage of colonies ± SEM of three independent experiments, each performed in duplicate. ***P < 0.001. (D) M74‐cells overexpressing wild‐type KLK7 (M74‐H), or its active site mutant (M74‐KLK7‐S/A), or M74‐vector cells (M74‐mock) were plated as in (C). Columns represent the mean percentage of colonies ± SEM of three independent experiments, each performed in duplicate. M74‐H *versus* M74‐mock, ***P <* *0.001; M74‐KLK7‐S/A *versus* M74‐mock, NS (*P* > 0.05).

Although previous studies have suggested that KLK7 acts as a proliferative factor in a number of cancer types (Kryza *et al*., 2016; Stefanini *et al*., 2015; Walker *et al*., 2014; Xi *et al*., 2015), in the M74‐D6 and M74‐H melanoma cell lines, KLK7 overexpression surprisingly suppressed cell growth compared to M74‐mock cells (Fig. 6A). M74‐transfected cells expressing a KLK7 mutant (KLK7‐S/A), where the catalytic serine residue is replaced by alanine (Fig. S1), did not show any significant differences in cell proliferation compared to vector control cells (Fig. 6B). Similarly, in a colony formation assay, both the M74‐D6 clone and the batch‐transfected M74‐H cells showed a significant decrease in the number of colonies when compared to M74‐mock cells (Fig. 6C). Again, the colony numbers of the mutant M74‐KLK7‐S/A cells did not differ from those obtained with vector control cells (Fig. 6D). These findings indicate that increased enzymatic activity of KLK7 reduces melanoma cancer cell proliferation and colony formation.

### 3.6 KLK7 overexpression alters cell morphology and modulates expression of cell adhesion molecules

**Figure 7:**
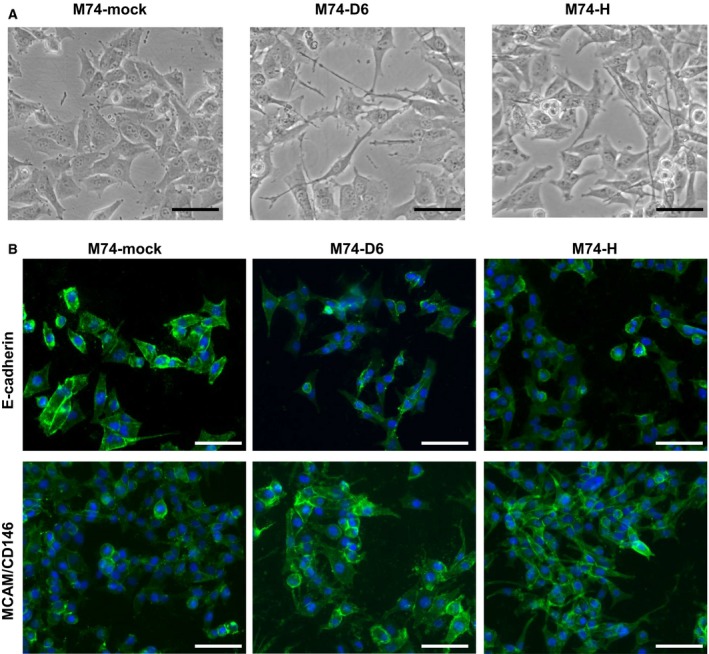
KLK7 overexpression in M74 cells results in altered cell morphology and modulates expression of cell adhesion molecules. (A) Representative phase‐contrast images of KLK7‐overexpressing cells: both the clonal M74‐D6 and the batch‐transfected M74‐H KLK7 overexpressing cell lines reveal an altered cell morphology compared with M74‐mock vector control cells. Cells overexpressing KLK7 display a spindle‐shaped morphology with membrane protrusions. Scale bar = 50 μm. (B) Representative immunofluorescence images (green) show a strong decrease in E‐cadherin (upper panel) and increase in MCAM/CD146 (lower panel) staining in KLK7‐overexpressing M74‐D6 and M74‐H cells compared to M74‐mock cells. Nuclear staining was performed with DAPI (blue). Scale bar = 100 μm.

Strikingly, both M74‐D6 and M74‐H cells overexpressing KLK7 displayed an elongated and irregular morphology with membrane protrusions typical of invading tumor cells not seen in M74‐mock cells (Fig. 7A). As KLK7 has been implicated in the shedding of adhesion molecules (Johnson *et al*., 2007; Ramani *et al*., 2008), we analyzed E‐cadherin expression in KLK7‐overexpressing cells *versus* vector control cells, applying an antibody that recognizes the extracellular region of E‐cadherin. As shown in Fig. 7, E‐cadherin staining was markedly decreased in M74‐D6 and M74‐H cells, as indicated by a weak and diffuse staining, whereas a strong membrane‐associated staining of E‐cadherin was observed in the M74‐mock cells (Fig. 7B, upper panel).

MCAM/CD146 (melanoma cell adhesion molecule) was originally identified as a marker of melanoma progression (Haass and Herlyn, 2005). Interestingly, MCAM/CD146 staining was significantly increased in KLK7‐overexpressing M74‐D6 and M74‐H cells compared to M74‐mock cells (Fig. 7B, lower panel). These results suggest that KLK7 induces downregulation of cell adhesion molecules (such as E‐cadherin), thus triggering a more motile and invasive phenotype accompanied with a concomitant upregulation of other adhesion molecules (such as MCAM/CD146) involved in homotypic cell adhesion and interaction with the extracellular matrix.

### 3.7 KLK7 overexpression supports melanoma cell migration and invasion

**Figure 8:**
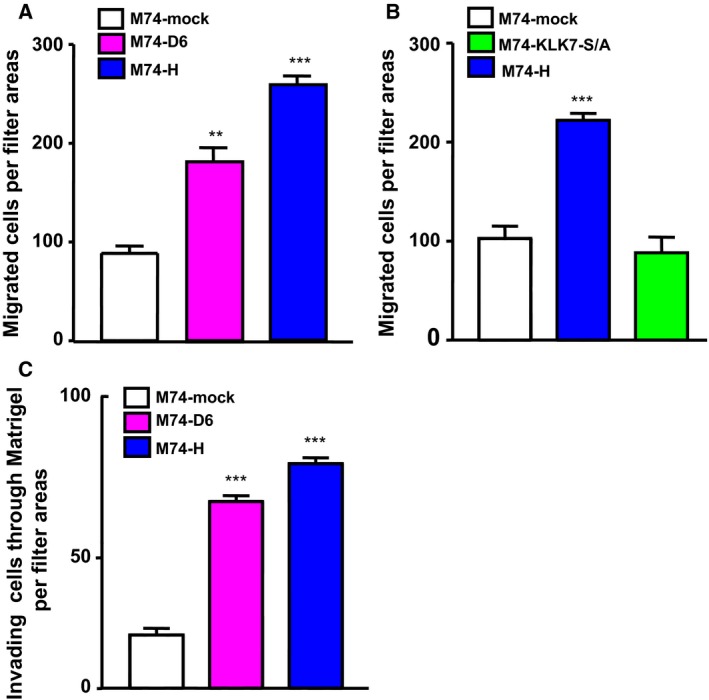
KLK7 overexpression but not KLK7 mutant induces melanoma cell migration and invasion. (A) M74‐D6, M74‐H, and M74‐mock cells were plated on 8‐μm pore‐size Transwell^®^ inserts. After 24 h, cells were stained with crystal violet/methanol and migrating cells were counted using an optical imaging system. Migrated cells from six filter fields were calculated with the image j software. **P <* *0.01; ***P <* *0.001. (B) M74‐cells overexpressing wild‐type KLK7 (M74‐H), its active site mutant (M74‐KLK7‐S/A), or M74‐vector cells (M74‐mock) were plated and analyzed as described in (A). M74‐H *versus* M74‐mock, ***P <* *0.001; M74‐KLK7‐S/A *versus* M74‐mock. NS (*P* > 0.05). (C) Transwell invasion assay: M74‐D6, M74‐H, and M74‐mock cells were plated on 8‐μm pore‐size Transwell^®^ inserts precoated with 10 μg of Matrigel™ (5 × 10^4^ per well) and invasion of the cells analyzed as described in (A). ***P < 0.001.

Because reduced E‐cadherin expression may reflect the gain of migratory properties and promotion of cell invasion, we investigated the effect of KLK7 expression on cell motility using a modified Boyden chamber assay. While KLK7 overexpression repressed cell proliferation, it interestingly induced a significant increase in cell migration in both M74‐D6 and M74‐H cells compared to M74‐mock cells (Fig. 8A). M74‐KLK7‐S/A cells, expressing catalytically inactive KLK7, did not show any significant differences in cell migration compared to vector control cells (Fig. 8B).

Next, cellular invasion was assessed using an *in vitro* Matrigel invasion assay to determine whether the increased migration rate induced by KLK7 in melanoma cells was associated with increased invasive properties as well. For this, cells were seeded into the upper chamber of a Matrigel™‐coated filter, and serum‐containing medium was used as chemoattractant in the lower chamber. Twenty‐four hours after seeding, cells that invaded through the Matrigel substrate were quantified on the lower side of the filter. As shown in Fig. 8C, KLK7‐overexpressing M74‐D6 and M74‐H cells revealed a strong and significant increase in cell invasion relative to vector control cells.

## 4 Discussion

One of our main findings is that several KLKs are ectopically expressed in skin melanomas compared to normal melanocytes, which do not express any of the *KLK* genes analyzed. Particularly, KLK7 was found to be the major secreted KLK in melanoma *in vitro* and was ectopically expressed *in vivo* in resected human melanoma tissues as well. In addition, melanoma cells that overexpress KLK7 exhibited a significant increase in cell migration and invasion. Thus, KLK7 may be an aberrantly expressed melanoma‐produced proteinase with tumorigenic functions and may represent a potential key element in melanoma progression. Although KLK7 has been described as a biomarker in many types of cancers (Devetzi *et al*., 2013; Kryza *et al*., 2016; Walker *et al*., 2014), to our knowledge, this is the first report demonstrating a role for KLK7 in the metastatic process of melanoma. Our study showed that only 69% of the analyzed melanoma cell lines ectopically express *KLK7*, whereas *KLK14* and *KLK6* are expressed by almost all of the cell lines. Nevertheless, when examined by ELISA, KLK7 was found to be the most abundantly secreted protein (up to 958 ng·L^−1^) into the culture media of the melanoma cells. The observed antigen levels were similar to those found in normal human epidermal keratinocytes (~ 1 μg·L^−1^) that constitutively express KLK7 (Hatano *et al*., 2013). *KLK7* expression was found both in primary melanoma cell lines (such as WM115, Dauv1, HM11) and in metastatic cell lines (such as SK‐Mel 5 and MT10). In papillary thyroid cancer, KLK7 upregulation was found to be correlated with BRAF mutations (Kim *et al*., 2010). Although BRAF, NRAS, or C‐Kit mutations have been identified as frequently mutated oncogenes in melanoma (Flaherty *et al*., 2012), no correlation was found between *KLK7* and the mutation status in our study (see Table S2). This suggests that KLK7 upregulation in melanoma may be rather influenced by other factors from the tumor microenvironment. In line with this hypothesis, KLK7 has been shown to be upregulated when benign hyperplastic prostate epithelial cells were cocultured with fibroblasts but not in separately cultured epithelial cells (Yang *et al*., 2010).

As demonstrated by immunohistochemistry, our analysis clearly shows that KLK7 is highly expressed in both primary melanoma and metastatic melanoma tissues, but only low KLK7 levels, if at all, are seen in nevi or in the normal melanocytes in the healthy skin, from which these cancers may arise. This is in line with the failure to detect any KLK7 mRNA in primary NHEM by PCR. The melanoma tumor specimens were only investigated by immunohistochemical techniques and not by RT‐PCR because the melanoma specimens contain keratinocytes of the stratum corneum and stratum granulosum, which are a major source of KLK7 (Egelrud *et al*., 2005; Yousef *et al*., 2000). Interestingly, we observed a significant trend between KLK7 staining intensity and the severity of the disease. The percentage of cells displaying strong staining intensity (13.5%) or moderate (25%) was higher in metastatic melanomas compared to primary melanomas (only 3.5% and 18%, respectively). Therefore, KLK7 might be considered as a potential progression marker for skin melanoma. In concordance with our findings, a previous gene expression study revealed that *KLK7, KLK4,* and *KLK11* expression is correlated with metastatic dissemination and associated with overall survival of patients with primary cutaneous melanoma (Winnepenninckx *et al*., 2006). In our study, only one benign nevus (of six) weakly stained for KLK7. Whether the weak KLK7 staining could display clinical significance (i.e., detection of early melanocytic transformation) is not yet clear but deserves to be further explored. Interestingly, KLK7 has been found to be a useful biomarker to distinguish various types of kidney tumors when morphology is similar (Gabril *et al*., 2010).

In line with our detection pattern, more recently, KLK7 expression has been found to be increased in atypical nevi and primary melanoma compared to its expression in common nevi (Martins *et al*., 2011; Rezze *et al*., 2011). However, the study by Martins and coworkers did not show significant differences between KLK7 staining in primary and metastatic tissues (Martins *et al*., 2011). The differences concerning KLK7 staining intensities in metastatic tissues *versus* primary melanoma in our study and in the study of Rezze *et al*. may be due to the sensitivity/specificity of the techniques used for detection, statistical analysis methods, and/or the specimen selection (cohorts). Of note, Rakosy *et al*. (2013) found that KLK7 was downregulated in ulcerated primary melanoma compared with nonulcerated tissues. The occurrence of KLK7 mRNA variants could also explain the existence of different expression patterns. Indeed, it has been reported that some KLKs including KLK7 use alternative promoters to generate organ‐ and disease‐specific transcripts (Dong *et al*., 2008).

Some KLKs have been shown to signal through multiple pathways to stimulate cell proliferation. (Chung *et al*., 2012; Filippou *et al*., 2015; Gratio *et al*., 2010, 2011; Kryza *et al*., 2016). KLK7 involvement in cell growth has also been demonstrated in different cancers. Indeed, simultaneous expression of KLK4, KLK5, KLK6, and KLK7 in ovarian cancer cells resulted in an increase in tumor burden *in vivo* (Prezas *et al*., 2006), KLK7 increased esophageal adenocarcinoma cell proliferation (Xi *et al*., 2015), and we have recently revealed that in colon cancer cells overexpressing KLK7 proliferation was increased *in vitro* and, moreover, these cells developed larger tumors in nude mice (Walker *et al*., 2014). Surprisingly, in the present study, we found that KLK7 overexpression strongly suppressed cell proliferation and colony formation of melanoma cells *in vitro*. The observed inhibitory effect on cell proliferation suggests that KLK7 may exert opposite effects depending on the cancer cell type. The KLK7‐mediated reduction in proliferation may suggest a common mechanism shared with some other KLKs under certain conditions, which also trigger antiproliferative effects in prostate cancer (Veveris‐Lowe *et al*., 2005), ovarian cancer (Pepin *et al*., 2011; Prezas *et al*., 2006), and breast cancer cells (Sotiropoulou *et al*., 2009).

As previous studies have shown that KLK7 mediates both proteolytic and nonproteolytic functions in ovarian cancer (Dong *et al*., 2014; Kryza *et al*., 2016), we used an enzymatically inactive KLK7 mutant, where the active site serine residue has been replaced by alanine. The mutant KLK7‐S/A did not affect cell proliferation and did not induce cell migration (Figs 6B, D and 8B), suggesting that KLK7 effects are mostly mediated by its proteolytic activity. In cell biological experiments, we were unable to directly follow the enzymatic activity of secreted KLK7 into the cell media, most likely because of the low enzyme concentration (≈ 38 pm, Fig. 2). Thus, the produced KLK7 levels seem sufficient to mediate cell responses (cell migration and cell invasion) but not to detect KLK7 enzymatic activity using fluorogenic chymotrypsin substrates, which require much higher enzyme concentrations in the nanomolar range (data not shown). To enable studies of the enzymatic activity of proteases involved in cell signaling, new highly sensitive tools have to be developed. Nevertheless, the KLK7 axis, which depends on enzymatic activity, represents a promising target and calls for further *in vivo* studies and also for the development of selective inhibitors of KLK7.

The mechanism by which KLK7 inhibits cell proliferation is not clear but may be due to cell cycle arrest or processing of repressive factors (cytokines) such as IL‐1beta, a known substrate of KLK7 (Nylander‐Lundqvist and Egelrud, 1997) that has been shown to inhibit melanoma growth (Neville *et al*., 1990). Accumulating evidence indicates that EMT, often accompanied by loss of E‐cadherin expression, plays a critical role in metastasis, whereby the genes involved in EMT promote cell migration and invasion but suppress proliferation of melanoma (Haass and Herlyn, 2005; Pearlman *et al*., 2017). In pancreatic cells, KLK7 induced shedding of E‐cadherin (Johnson *et al*., 2007) and induced EMT in prostate cancer (Mo *et al*., 2010) associated with a remarkably increased cell migration and invasion. In our study, we found that KLK7 overexpression induced changes in cell morphology and a significant decrease in E‐cadherin associated with an increase in MCAM/CD146 expression. Melanoma cells do not show a classical epithelial or mesenchymal phenotype (Kim *et al*., 2013). In fact, analysis of EMT markers did not reveal significant differences in vimentin and N‐cadherin expression in M74‐control cells and KLK7‐overexpressing cells (Fig. S4). These data suggest that the observed KLK7‐mediated decrease in E‐cadherin is not due to an EMT process but may be due to cleavage/degradation of the extracellular domain by the secreted KLK7. The KLK7‐mediated loss of E‐cadherin and gain in MCAM/CD146 may suggest a possible role of KLK7 in melanoma cell aggregation, a known mechanism of chemoresistance in cancer.

Although cell proliferation is the hallmark of most cancers, invasion and proliferation are uncoupled in melanoma, such that highly proliferative melanoma cells are less likely to be invasive, and *vice versa*, a phenomenon called ‘phenotype switching’ (Hoek *et al*., 2008). It has been suggested that tissue microenvironmental factors signal the switch of some cells to an invasive phenotype. Thus, in addition to genetic heterogeneity, acquired phenotype heterogeneity contributes to therapy failure with the currently used MAP kinase pathway inhibitors. To identify the role of KLK7 in melanoma progression, we measured the *in vitro* invasive potential. Our results showed that KLK7 overexpression led to a more migratory and more invasive phenotype. This observed KLK7‐induced effect on the dissemination process is consistent with the described role of KLK7 in cell invasion (Johnson *et al*., 2007; Mo *et al*., 2010; Prezas *et al*., 2006) and its association with unfavorable prognosis in several types of cancers (Dong *et al*., 2010; Kryza *et al*., 2016).

The mechanisms underlying the KLK7‐mediated increase in cell motility and invasion remain unknown but might be directed by modulating other enzymatic pathways important in cancer metastasis. Previous studies showed that KLK7 also cleaved pro‐MMP9 generating active MMP9 (Ramani *et al*., 2011), and uPAR (Ramani and Haun, 2008), two members of key proteolytic systems in melanoma invasion (Frohlich, 2010). However, it is unlikely that MMP9 is involved in the KLK7 effect seen in our studies, as analysis of protease array data as well as western blot analysis using conditioned media from control M74 cells and from KLK7‐transfected M74 cells failed to detect any MMP9 protein (data not shown). Although the pathways responsible for KLK7‐mediated stimulation of melanoma cell migration and invasion are beyond the scope of this report, it is worth to note that using protease array techniques, an upregulation of members of the cathepsin family, certain matrix metalloproteinases, and proteases inhibitors was observed in cell culture supernatants of melanoma cells that overexpress KLK7 (data not shown). Because proteases often act indirectly by inducing other proteases or other factors, more global approaches such as arrays and/or proteomic, peptide mapping, and degradomic analysis may have to be applied in order to identify KLK7 substrates or specific pathways that are activated by KLK7.

In conclusion, we have shown that KLK7 is an aberrantly expressed melanoma‐produced proteinase and that KLK7 decreases cell proliferation and colony formation but induces an increase in cell motility and cell invasion. Our results, together with those from previous reports, indicate a correlation of KLK7 expression with melanoma metastasis and suggest that KLK7 is a potential biomarker for melanoma progression.

## Author contributions

DD was involved in study conception and design and acquired the data. TD, MH, SA, FS, and DD provided technical support and access to equipment. MB, CEA, FS, VM and EPD provided material support. LD, FW, VM, and DD analyzed and interpreted the data. VM and DD wrote the manuscript. DD, VM, LD, FW, and MB reviewed and revised the manuscript.

## Supporting information


**Fig. S1.** Sequence of KLK7‐S/A in pRcRSV.

**Fig. S2.** QPCR analysis of KLK7 mRNA expression in a subset of melanoma cell lines and in normal melanocytes.

**Fig. S3.** KLK7 triggers p42/p44 MAP kinase (ERK1/2) but not Smad2 or Stat3 phosphorylation in MeWo melanoma cells.

**Fig. S4.** Analysis of EMT expression markers in KLK7‐overexpressing cells.

**Table S1.** Primer sets used for RT‐PCR, the expected amplicons (bp) and annealing temperature.

**Table S2.** Melanoma cell lines and mutational status in malignant melanoma.
