# Supplementary material for: Aberrant expression of kallikrein‐related peptidase 7 is correlated with human melanoma aggressiveness by stimulating cell migration and invasion
Source: Mol Oncol. 2017 Aug 11;11(10):1330–47. doi: 10.1002/1878-0261.12103 (PMC5623816; doi:10.1002/1878-0261.12103)
Supplement: Supplementary file 1 — Fig. S1. Sequence of KLK7‐S/A in pRcRSV. [file MOL2-11-1330-s001.pdf]

Figure S1: Sequence of KLK7-S/A in pRcRSV

```
  1 atggcaagatcccttctcctgcccctgcagatcctactgctatcc
     M  A  R  S  L  L  L  P  L  Q  I  L  L  L  S

 46 ttagccttggaaactgcaggagaagaagcccagggtgacaagatt
     L  A  L  E  T  A  G  E  E  A  Q  G  D  K  I

 91 attgatggcgccccatgtgcaagaggctcccacccatggcaggtg
     I  D  G  A  P  C  A  R  G  S  H  P  W  Q  V

136 gccctgctcagtggcaatcagctccactgcggaggcgtcctggtc
     A  L  L  S  G  N  Q  L  H  C  G  G  V  L  V

181 aatgagcgctgggtgctcactgccgcccactgcaagatgaatgag
     N  E  R  W  V  L  T  A  A  H  C  K  M  N  E

226 tacaccgtgcacctgggcagtgatacgctgggcgacaggagagct
     Y  T  V  H  L  G  S  D  T  L  G  D  R  R  A

271 cagaggatcaaggcctcgaagtcattccgccaccccggctactcc
     Q  R  I  K  A  S  K  S  F  R  H  P  G  Y  S

316 acacagacccatgttaatgacctcatgctcgtgaagctcaatagc
     T  Q  T  H  V  N  D  L  M  L  V  K  L  N  S

361 caggccaggctgtcatccatggtgaagaaagtcaggctgccctcc
     Q  A  R  L  S  S  M  V  K  K  V  R  L  P  S

406 cgctgcgagccccctggaaccacctgtactgtctccggctggggc
     R  C  E  P  P  G  T  T  C  T  V  S  G  W  G

451 actaccacgagcccagatgtgacctttccctctgacctcatgtgc
     T  T  T  S  P  D  V  T  F  P  S  D  L  M  C

496 gtggatgtcaagctcatctcccccaggactgcacgaaggtttac
     V  D  V  K  L  I  S  P  Q  D  C  T  K  V  Y

541 aaggacttactggaaaattccatgctgtgcgctggcatccccgac
     K  D  L  L  E  N  S  M  L  C  A  G  I  P  D

586 tccaagaaaaacgcctgcaatggtgacgccggcggaccgttggtg
     S  K  K  N  A  C  N  G  D  A  G  G  P  L  V

631 tgcagaggtaccctgcaaggtctggtgtcctggggaactttccct
     C  R  G  T  L  Q  G  L  V  S  W  G  T  F  P

676 tgcggccaacccaatgacccaggagtctacactcaagtgtgcaag
     C  G  Q  P  N  D  P  G  V  Y  T  Q  V  C  K

721 ttcaccaagtggataaatgacaccatgaaaaagcatcgctaa
     F  T  K  W  I  N  D  T  M  K  K  H  R  *
```
