# Supplementary figures and images for: Aberrant expression of kallikrein‐related peptidase 7 is correlated with human melanoma aggressiveness by stimulating cell migration and invasion
Source: Mol Oncol. 2017 Aug 11;11(10):1330–47. doi: 10.1002/1878-0261.12103 (PMC5623816; doi:10.1002/1878-0261.12103)

**Figure S2**

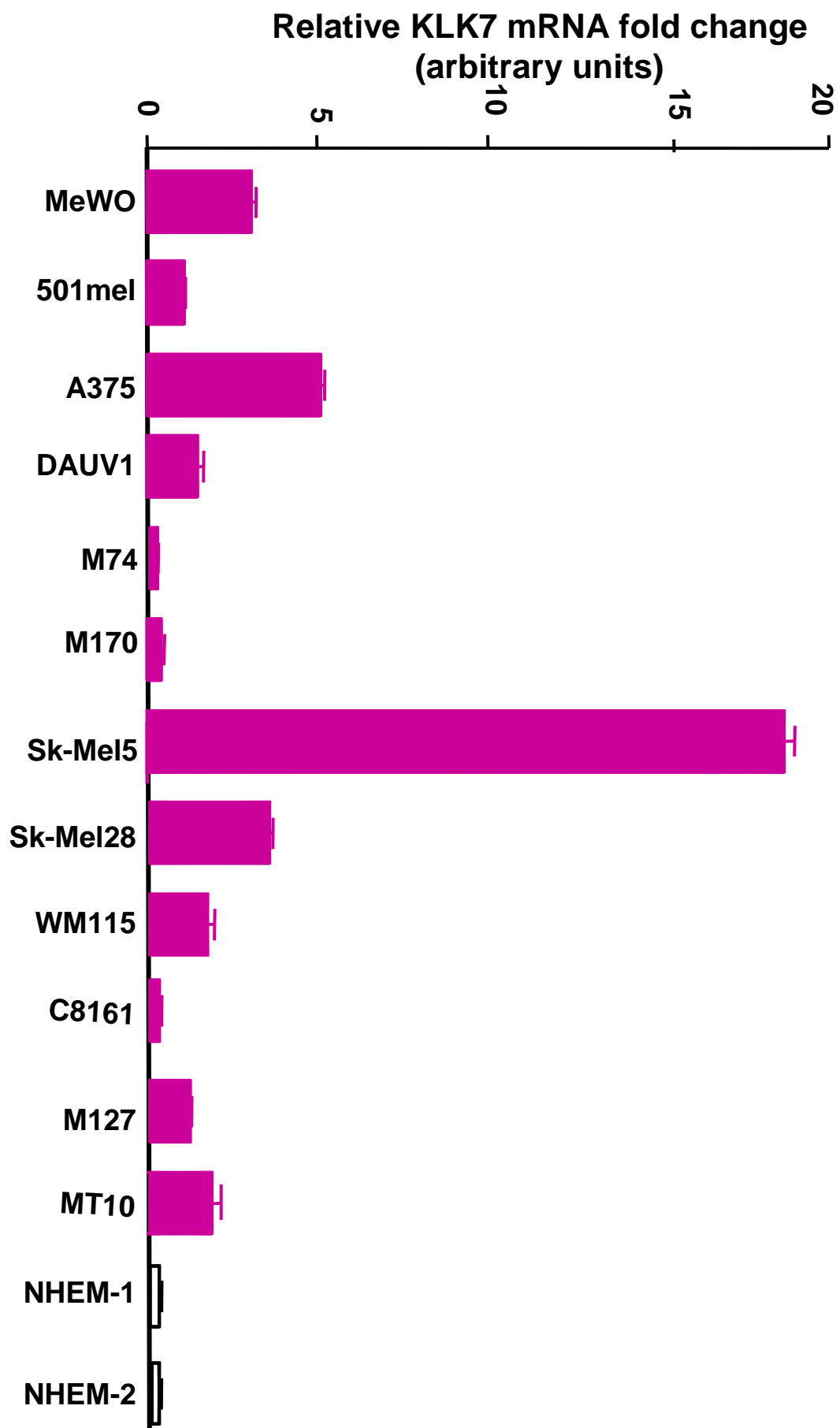

Supplement: Supplementary file 2 — Fig. S2. QPCR analysis of KLK7 mRNA expression in a subset of melanoma cell lines and in normal melanocytes. [file MOL2-11-1330-s002.pdf]

**A**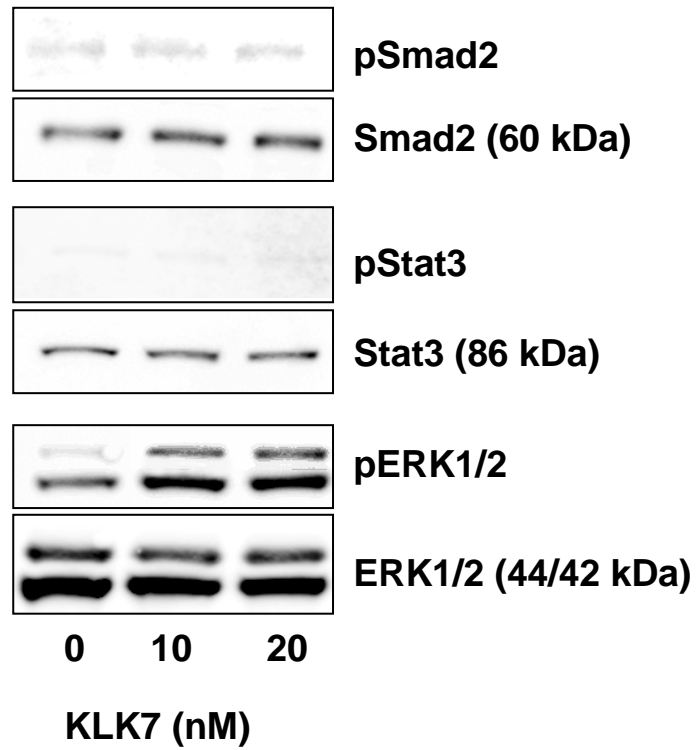**B**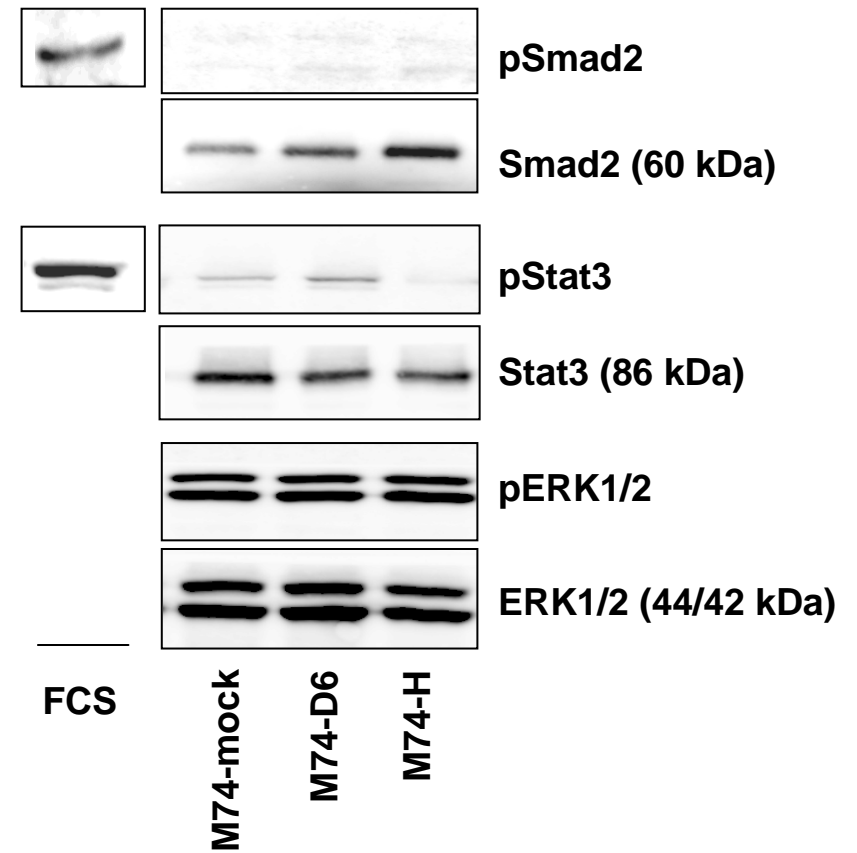**Figure S3**

Supplement: Supplementary file 3 — Fig. S3. KLK7 triggers p42/p44 MAP kinase (ERK1/2) but not Smad2 or Stat3 phosphorylation in MeWo melanoma cells. [file MOL2-11-1330-s003.pdf]

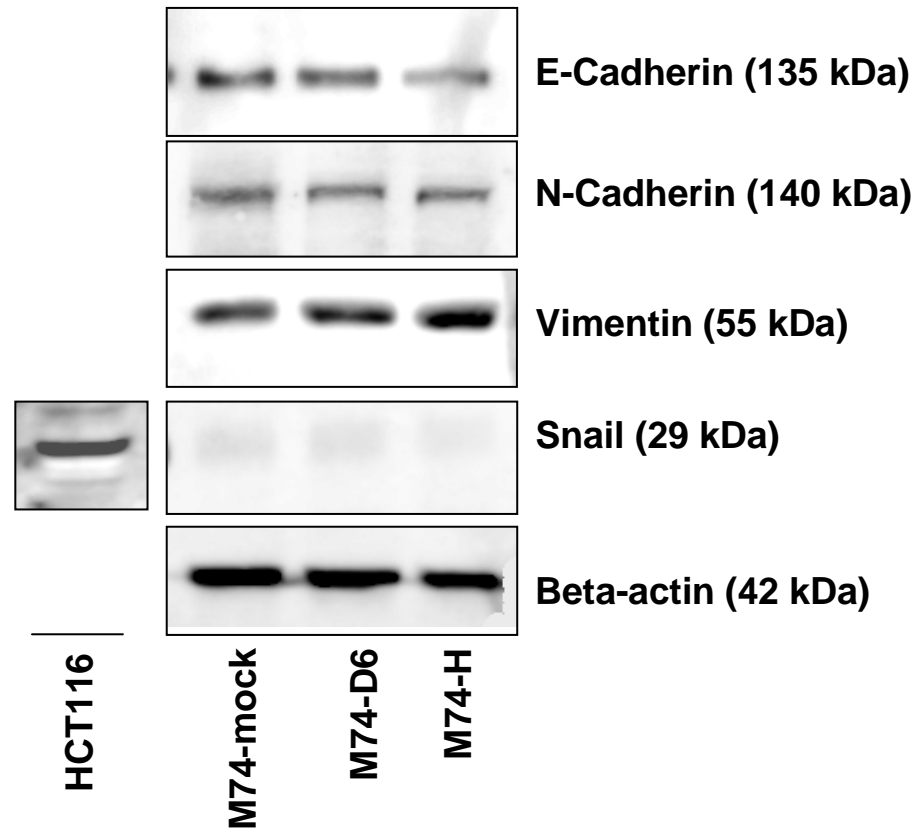

**Figure S4**

Supplement: Supplementary file 4 — Fig. S4. Analysis of EMT expression markers in KLK7‐overexpressing cells. [file MOL2-11-1330-s004.pdf]
