# Supplementary material for: Aberrant expression of kallikrein‐related peptidase 7 is correlated with human melanoma aggressiveness by stimulating cell migration and invasion
Source: Mol Oncol. 2017 Aug 11;11(10):1330–47. doi: 10.1002/1878-0261.12103 (PMC5623816; doi:10.1002/1878-0261.12103)
Supplement: Supplementary file 5 — Table S1. Primer sets used for RT‐PCR, the expected amplicons (bp) and annealing temperature. [file MOL2-11-1330-s005.pdf]

Table S1 : Primer sets used for RT-PCR , the expected amplicons (bp) and annealing temperature

mRNAs	Primers	Product length (bp)	Annealing temperature (°C)
KLK 4	Forward : 5′- GGATCGCTCGTCTCTGGTAG-3′	561	60
	Reverse: 5′-AGAGTCACCGTTGCAGGAG-3′		
KLK 5	Forward : 5’-CAAGACCCCCCTGGATGTGG-3’	345	65
	Reverse : 5’-CCGAGACGGACTCTGAAAACTTTCTTCC-3’		
KLK 6	Forward : 5’GAAGCTGATGGTGGTGCTGAGTCTG	454	61
	Reverse : 5’AG ACAGCAGATGGTGATTTCCCTGAC		
KLK 7	Forward: 5’-GCCCAGGGTGACAAGATTATT-3’	569	62
	Reverse : 5’-GTACCTCTGCACACCAACGG-3’		
KLK 8	Forward : 5′-TACTCTGTGGCGGTGTCCTTG-3′	523	63
	Reverse: 5′-GAGCCCCAGGATGTGATGCCC-3′		
KLK 10	Forward : 5′- GCGGAAACAAGCCACTGTGGG -3′	486	60
	Reverse: 5′-GGTAAACACCCCACGAGAGGA-3′.		
KLK 14	Forward : 5′-CACTGCGGCCGCCCGATC-3′	485	65
	Reverse: 5′-GGCAGGGCGCAGCGCTCC-3′		
GADPH	Forward : 5’-TCGGAGTCAACGGATTTGGTCGTA-3’	305	65
	Reverse : 5’-AGCCTTCTCCATGGTGGTGAAGA-3’		
