# Supplementary material for: Aberrant expression of kallikrein‐related peptidase 7 is correlated with human melanoma aggressiveness by stimulating cell migration and invasion
Source: Mol Oncol. 2017 Aug 11;11(10):1330–47. doi: 10.1002/1878-0261.12103 (PMC5623816; doi:10.1002/1878-0261.12103)
Supplement: Supplementary file 6 — Table S2. Melanoma cell lines and mutational status in malignant melanoma. [file MOL2-11-1330-s006.pdf]

Table S2 *:* Melanoma cell lines and mutational status in malignant melanoma

Cell line	Mutation	phenotype
Colo 792	WT	Metastatic melanoma
MEWO		Metastatic melanoma
501 Mel	^{V600E} BRAF	Metastatic melanoma
A375		Primary melanoma
Colo 829		Primary melanoma
Colo 794		Metastatic melanoma
Dauv1		Primary melanoma
M74		Metastatic melanoma
MM170		Metastatic melanoma
SK-Mel5		Metastatic melanoma
SK-Mel28		Primary melanoma
WM115	^{V600D} BRAF	Primary melanoma
WM266-4		Metastatic melanoma
C8161	^{Q61K} NRAS	Metastatic melanoma
HM11		Primary melanoma
Sbcl2		Primary melanoma
WM1361		Primary melanoma
WM852	^{Q61R} NRAS	Metastatic melanoma
XP44RO	^{Q61H} NRAS	Metastatic melanoma
MM127	^{G13R} NRAS	Metastatic melanoma
MT10	^{Q61R} NRAS + INS27b	Metastatic melanoma
M230	^{L576P} KIT	Metastatic melanoma
WM1791c	^{Q61H} KRAS	Metastatic melanoma

(Dumaz et al., 2006), Web site: https://www.wistar.org/lab/meenhard-herlyn-dvm-dsc/page/melanoma-cell-lines-0
